# Depression and Advance Care Planning Among Japanese Patients Undergoing Hemodialysis: Japanese Dialysis Outcomes and Practice Pattern Study (J-DOPPS)

**DOI:** 10.1016/j.xkme.2025.101210

**Published:** 2025-12-12

**Authors:** Hiroki Nishiwaki, Chisato Miyakoshi, Jun Miyashita, Yoshihiro Onishi, Noriaki Kurita, Hirotaka Komaba, Ken Sakai

**Affiliations:** 1 Department of Clinical Epidemiology, Graduate School of Medicine, Fukushima Medical University, Fukushima, Japan; 2 Division of Nephrology, Department of Medicine, Showa Medical University Fujigaoka Hospital, Yokohama, Japan; 3 Department of Pediatrics and Neonatology, Kobe City Medical Center General Hospital, Kobe, Japan; 4 Department of Healthcare Epidemiology, School of Public Health in the Graduate School of Medicine, Kyoto University, Kyoto, Japan; 5 Department of Research Support, Center for Clinical Research and Innovation, Kobe City Medical Center General Hospital, Kobe, Japan; 6 Department of General Medicine, Shirakawa Satellite for Teaching And Research (STAR), Fukushima Medical University, Shirakawa, Japan; 7 Institute for Health Outcomes & Process Evaluation Research, Kyoto, Japan; 8 Department of Innovative Research and Education for Clinicians and Trainees (DiRECT), Fukushima Medical University Hospital, Fukushima, Japan; 9 Division of Rheumatology, Department of Medicine, Showa Medical University School of Medicine, Tokyo, Japan; 10 Division of Nephrology, Endocrinology and Metabolism, Tokai University School of Medicine, Isehara, Japan; 11 Department of Nephrology, Toho University Faculty of Medicine, Tokyo, Japan

**Keywords:** Advance care planning, depression, hemodialysis

## Abstract

**Rationale & Objective:**

Advance care planning (ACP) is crucial in end-of-life care. Data on ACP discussion among patients with end-stage kidney disease are limited. One study has suggested that depressive symptoms increase ACP discussion.

**Study Design:**

This study aimed to analyze the association between depression and ACP discussion in patients undergoing hemodialysis.

**Setting & Population:**

This used data from the Japan Dialysis Outcomes and Practice Patterns Study.

**Predictor:**

Both cross-sectional and longitudinal associations between depressive symptoms and ACP discussion were examined.

**Outcomes:**

Depressive symptoms were defined as a score of ≥10 points on the 10-item Center for Epidemiologic Studies Depression scale. ACP discussion was defined as discussing ACP with health care providers and family members.

**Analytical Approach:**

Generalized estimating equations and generalized linear models based on Poisson distribution and log-link function were used to estimate prevalence (PR) and incidence proportion ratios (IPRs) using robust standard errors, respectively.

**Results:**

Data in 2016 and 2017 included 2,443 patients for the cross-sectional analysis and 870 for the longitudinal analysis. ACP discussion was 26% in 2016 and 28% in 2017, with depressive symptoms rates of 45% and 47%, respectively. The cross-sectional analysis indicated a positive association between depressive symptoms and ACP discussion (adjusted PR, 1.20; 95% confidence interval (CI), 1.05-1.37). Depressive symptoms were not significantly associated with ACP discussion in longitudinal analyses (adjusted IPR, 1.10; 95% CI 0.80-1.51).

**Limitations:**

Sample size, unadjusted confounding, and generalizability across cultural backgrounds.

**Conclusions:**

Our study showed an association between depressive symptoms and ACP in the cross-sectional analysis, but not longitudinally.

## Plain-Language Summary

Thinking ahead about medical decisions is vital for people receiving dialysis, yet we know little about how often these conversations happen or what drives them. We analyzed national survey data from Japanese dialysis centers to see whether feelings of depression encourage or discourage patients from talking with their families and care teams about future care. We compared patients at one point in time and again 1 year later. At baseline, those reporting depressive feelings were more likely to have had these planning talks, but this link did not hold over the following year. Our work suggests that recognizing emotional distress may open a useful doorway to end-of-life discussions and better patient-centered care for dialysis patients and their supportive networks today.

Advance care planning (ACP) in end-of-life (EoL) care ensures that patients receive patient-centered care even when communication is difficult. ACP takes into account patients’ wishes that have been expressed to the family and others. It can reduce the suffering of patients and their families, as well as the emotional distress of health care professionals.^1^^,^^2^

Despite the importance of ACP in kidney failure with replacement therapy (KFRT), with its high mortality rate, the frequency of ACP discussion has been noted to be generally not high. KFRT is a major chronic illness, with an estimated incidence rate of 396 cases per million people in the United States and 307 cases per million people in Japan.^3^^,^^4^ Therefore, ACP is important for patients with KFRT. Identifying facilitators and barriers to ACP discussion is urgent, given that approximately 26% of patients with KFRT are reported to have discussed their intention for EoL care with their dialysis physicians.^5^ Older age, malignancy, marital status, higher education, religious beliefs, and patient-centered care are known facilitators of ACP.^5^^,^^6^ In contrast, low literacy levels among patients and a lack of opportunities to discuss EoL care for both patients and practitioners are known disincentives for ACP.^7^^,^^8^ However, how psychological factors in patients undergoing dialysis, such as depressive symptoms, affect their ACP discussion has not been adequately investigated.

One cross-sectional study in primary care found that depressive symptoms were associated with higher ACP discussion and lack of interest in life extension.^9^ The authors argued that patients with depressive symptoms and anxiety may be more motivated to engage in ACP because they have made clear decisions regarding medical care in past circumstances. One study among patients receiving hemodialysis (HD) indicated that depressive symptoms may increase death anxiety.^10^ On the other hand, impaired decision-making capacity resulting from depressive symptoms (especially in severe cases) may lead to inappropriate decisions regarding life-extending treatment.^11^ Given the above, patients with HD and depressive states are expected to be more likely to engage in ACP because of their awareness of death. However, the association between depressive symptoms and ACP in patients with HD has as yet not been investigated, nor has the influence of ACP discussion on depressive symptoms (ie, reverse causation). Considering the high prevalence of depressive symptoms among patients receiving HD, addressing these unresolved issues is imperative.^10^^,^^12^

We hypothesized that depressive symptoms would be associated with ACP discussion as well as with life-extending therapies among patients with HD. To test this hypothesis, using data from the Japanese Dialysis Outcomes and Practice Pattern Study (J-DOPPS), we conducted 2 analyses: a cross-sectional analysis to examine whether depressive symptoms are associated with ACP discussion and a longitudinal analysis to verify the robustness of the temporal association using a subset of the data.

## Materials and Methods

### Design, Setting, and Participants

We examined the association between depressive symptoms and engaging in ACP, both cross-sectionally and longitudinally, using data from the J-DOPPS phase 6 conducted between 2015 and -2017. DOPPS (http://www.dopps.org) is an international prospective cohort study of HD practices that began in 1996. At the start of each study phase, DOPPS enrolled random samples of patients from stratified, national random samples of dialysis facilities, with departing patients being replaced as previously described.^13^^,^^14^ The J-DOPPS phase 6 participating sites consisted of 29 dialysis clinics and 27 hospitals. The protocol of J-DOPPS phase 6 was approved by the Ethics Committee of Tokyo Women’s Medical University (approval numbers: 2388-R4), and written informed consent was obtained from all participants.

We excluded patients without data related to depressive symptoms, as well as those without a history of dementia and those with missing responses regarding a history of dementia. Patients who provided unreliable responses to the questionnaire used to assess depressive symptoms were excluded.

### Exposure Measures

The main exposure was depressive symptoms, which were measured using the Japanese version of the 10-item Boston Form of the Center for Epidemiologic Studies Depression scale (CES-D).^15^ This test is routinely administered to all J-DOPPS participants as part of the core patient questionnaire. We adopted the CES-D in this study because of its high sensitivity in capturing a broad range of depressive symptoms and its well-established reliability and validity in epidemiological research. The CES-D has been validated in hemodialysis populations (sensitivity, 0.71; specificity, 0.83; Cronbach’s α, 0.80-0.85) and captures a broad range of depressive symptoms with high specificity.^16^, ^17^, ^18^ Presence of depressive symptoms was defined as a CES-D score of 10 or higher.^19^

The shorter form of the CES-D comprised 10 items, each scored on a 4-point Likert scale. Patients selected one of the following responses for each item: “less than 1 day,” (0 points) “1-2 days,” (1 point) “3-4 days,” (2 points) or “5-7 days” (3 points). The scale consisted of 2 types of statements: direct and reverse. Direct statements express negative feelings, whereas reversed items convey positive feelings. For instance, “I felt depressed” is a direct item, whereas “I was happy” is a reversed item. Responses were deemed inconsistent and unreliable if “less than 1 day” or “5-7 days” was selected for all items on CES-D, regardless of whether they were direct or reversed. Such cases were excluded from the analyses using regression models. The CES-D score was included in the models as a dichotomous variable based on the aforementioned cutoff.^19^

### Outcome Measures

The main outcome of this study was ACP discussion. Previous studies have indicated that ACP, combined with both formal advance directives and informal discussions, is more effective than completion of advance directives alone.^20^^,^^21^ Advance directives are rarely completed without informal ACP discussions. Given these findings and the absence of legally established advance directives in Japan, this study focused on ACP discussions with family members, family physicians, or both, in line with previous research.^22^ Therefore, our primary outcome was “having an ACP discussion,” defined as discussing ACP with health care professionals and family members, regardless of whether the conversation was subsequently documented in writing.^22^, ^23^, ^24^ In this study, ACP was determined by responses to the question suggested by O'Hare et al^25^: “Have you thought about the kinds of treatments you would want or not want if you were to become very sick and were unable to speak for yourself?”. The response options were as follows: “1. I have not thought about this.”, “2. I have thought about this, but have not talked to anyone about it.”, “3. I have talked about this with a friend or family member, but have not signed official papers.”, “4. I have talked about this with a doctor or other health care provider, but have not signed official papers” “5. I have signed official papers documenting my preferences (eg, living will or advance directives), but have not talked with any friends or family members about this.”, and “6. I have signed official papers documenting my preferences (eg, living will or advance directives), and have talked with at least one friend or family member about this”. We defined a choice of 3, 4, or 6 as “ACP discussed” and 6 as “advance directive”.

The secondary outcome of the study investigated wishes for life-prolonging treatment. The question regarding life-prolonging treatment was (1) “I want treatment that prolongs my life as much as possible,” which was defined as “opt for life-prolonging treatment” and compared with the group that answered the other options.

### Covariable Measures

Confounding variables were selected based on existing literature that indicated an association with either depressive symptoms or ACP.^9^ These variables included age, sex, dialysis vintage, comorbid conditions (such as cardiovascular disease, cerebrovascular disease, malignancy, and psychoneurotic disease including depression, bipolar disorder, schizophrenia, and dependence on alcohol or other substances within the past 12 months); household income (<3M, 3-5M, 5-8M, >8M yen, or unknown), marriage status (never married, married, widowed, divorced/separated, or unknown), educational background (less than 12 years, high school, college or university, graduate school, or unknown), and facility.

### Statistical Analysis

Continuous variables were reported as mean and standard deviation (SD), whereas categorical variables were presented as the total number and percentage.

To evaluate the association between depressive symptoms and ACP, we conducted both cross-sectional and longitudinal analyses.

In the cross-sectional analysis, we assessed the association between the CES-D scores and outcomes within the same year using cohorts from both 2016 and 2017. With this approach, data for the same patient could be extracted from 2 different years of data for analysis. Accordingly, to account for intraindividual correlations within a patient, we employed generalized estimating equations with an exchangeable correlation structure. Specifically, the adjusted prevalence ratios (PRs) were estimated based on robust standard errors by specifying a Poisson distribution with a log-link function.^26^

Using the 2016 survey as the baseline, we identified patients who had not yet engaged in an ACP discussion. Depressive symptoms (CES-D) measured at baseline served as the exposure, and new ACP discussions reported at the 2017 follow-up constituted the outcome, yielding a uniform 1-year risk period. Incidence proportion ratios (IPRs) were estimated using a Poisson regression (log link, robust standard errors), adjusting for baseline covariates and dialysis facility.

Age, sex, vintage, comorbid conditions (cerebrovascular disease, respiratory disease, cancer, and psychological disease, except for dementia), income, marital status, educational level, and facility identifier were included in the models as potential confounders.

Missing outcomes and covariates data were imputed using a fully conditional specification method, resulting in the creation of 100 complete datasets. Subsequent analyses were conducted separately for each of these datasets, and the results were combined using Rubin’s rule.

Statistical significance was defined as a *P* value < 0.05. All statistical analyses were performed using Stata MP 18.0 (Stata Corp, College Station, TX, USA) and R software, version 4.1.1 (R Foundation for Statistical Computing, Vienna, Austria).

## Results

**Figure 1:**
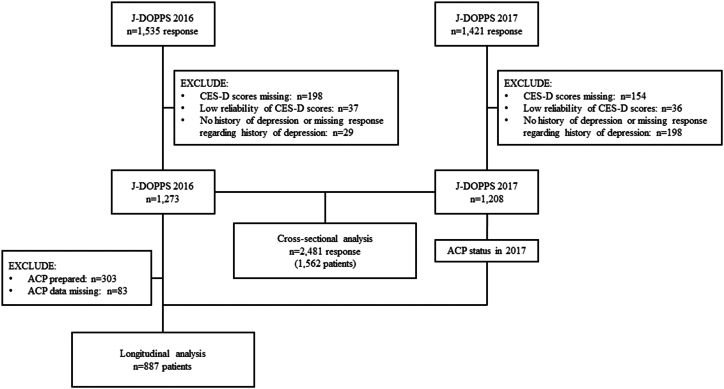
Patient flow chart. Abbreviations: ACP: Advance care planning; CES-D: Center for Epidemiologic Studies Depression scale; J-DOPPS: Japan Dialysis Outcomes and Practice Patterns Study.

Patients from the J-DOPPS phase 6 in 2016 and 2017 were included in the analysis (Fig 1). In the cross-sectional analysis, 1,273 responses in 2016 and 1,208 responses in 2017 were included, for a total of 2,481 responses from 1,562 patients in both years, and a total of 870 patients were included in the longitudinal study.

### Cross-sectional Analysis

Table 1 presents baseline data of the 2016 and 2017 cohorts according to the CES-D scores. In the 2016 cohort, 303 participants (26%) had engaged in ACP discussions, 36 (3.1%) had advance directives, and 41(3.4%) individuals had wishes for life-prolonging treatment. In the 2017 cohort, 323 participants (28%) engaged in ACP discussions, 59 (5.2%) had advance directives, and 39 individuals (3.4%) had wishes for life-prolonging treatment. The mean (SD) of the baseline CES-D scores were 9.7 (5.9) and 9.4 (5.8) in 2016 and 2017, respectively. In total, 591 (47%) and 537 patients (45%) had depressive symptoms in 2016 and 2017, respectively. In both years, most patients were aged 65-74 years, the sex distribution was 69% male, and years receiving dialysis were 1-5 years.

**Table 1:** Patient Characteristics for the Cross-sectional Analysis, Summarized by CES-D Score Groups

Patient characteristics	Year 2016			Year 2017		
	All	CES-D score		All	CES-D score	
		≤9	≥10		≤9	≥10
	n = 1,256	n = 665	n = 591	n = 1,187	n = 650	n = 537
Age						
≤54 y	253 (20%)	131 (20%)	122 (21%)	249 (21%)	130 (20%)	119 (22%)
55-64 y	328 (26%)	176 (26%)	152 (26%)	304 (26%)	180 (28%)	124 (23%)
65-74 y	449 (36%)	245 (37%)	204 (35%)	422 (36%)	237 (36%)	185 (34%)
75-84 y	201 (16%)	101 (15%)	100 (17%)	181 (15%)	89 (14%)	92 (17%)
≥85 y	25 (2.0%)	12 (1.8%)	13 (2.2%)	31 (2.6%)	14 (2.2%)	17 (3.2%)
Sex						
Female	391 (31%)	194 (29%)	197 (33%)	373 (31%)	201 (31%)	172 (32%)
Household income						
<3 M yen	558 (44%)	259 (39%)	299 (51%)	507 (43%)	252 (39%)	255 (47%)
3-5 M yen	271 (22%)	170 (26%)	101 (17%)	242 (20%)	155 (24%)	87 (16%)
5-8 M yen	128 (10%)	83 (12%)	45 (7.6%)	134 (11%)	85 (13%)	49 (9.1%)
>8 M yen	96 (7.6%)	63 (9.5%)	33 (5.6%)	99 (8.3%)	68 (10%)	31 (5.8%)
Unknown	203 (16%)	90 (14%)	113 (19%)	205 (17%)	90 (14%)	115 (21%)
Marital status						
Never married	197 (16%)	95 (14%)	102 (17%)	192 (16%)	90 (14%)	102 (19%)
Married	801 (64%)	443 (67%)	358 (61%)	745 (63%)	447 (69%)	298 (55%)
Widowed	114 (9.1%)	58 (8.7%)	56 (9.5%)	114 (9.6%)	54 (8.3%)	60 (11%)
Divorced/separated	102 (8.1%)	49 (7.4%)	53 (9.0%)	93 (7.8%)	38 (5.8%)	55 (10%)
Unknown	42 (3.3%)	20 (3.0%)	22 (3.7%)	43 (3.6%)	21 (3.2%)	22 (4.1%)
Educational background						
Less than 12 y	293 (23%)	126 (19%)	167 (28%)	273 (23%)	121 (19%)	152 (28%)
High school	512 (41%)	272 (41%)	240 (41%)	493 (42%)	271 (42%)	222 (41%)
College or university	155 (12%)	96 (14%)	59 (10.0%)	161 (14%)	99 (15%)	62 (12%)
Graduate school	249 (20%)	153 (23%)	96 (16%)	228 (19%)	142 (22%)	86 (16%)
Unknown	47 (3.7%)	18 (2.7%)	29 (4.9%)	32 (2.7%)	17 (2.6%)	15 (2.8%)
Duration of hemodialysis						
<1.0 y	270 (21%)	154 (23%)	116 (20%)	343 (29%)	199 (31%)	144 (27%)
1.0-5.0 y	418 (33%)	200 (30%)	218 (37%)	335 (28%)	169 (26%)	166 (31%)
5.0-10.0 y	243 (19%)	133 (20%)	110 (19%)	230 (19%)	129 (20%)	101 (19%)
>10.0 y	325 (26%)	178 (27%)	147 (25%)	279 (24%)	153 (24%)	126 (23%)
Comorbid condition						
Cardiovascular diseases	604 (48%)	305 (46%)	299 (51%)	563 (47%)	298 (46%)	265 (49%)
Cerebrovascular diseases	237 (19%)	117 (18%)	120 (20%)	210 (18%)	120 (18%)	90 (17%)
Respiratory diseases	85 (6.8%)	36 (5.4%)	49 (8.3%)	70 (5.9%)	35 (5.4%)	35 (6.5%)
Cancers	169 (13%)	98 (15%)	71 (12%)	157 (13%)	91 (14%)	66 (12%)
Psychiatric diseases	47 (3.7%)	14 (2.1%)	33 (5.6%)	45 (3.8%)	18 (2.8%)	27 (5.0%)
ACP status						
ACP discussion	303 (26%)	159 (25%)	144 (26%)	323 (28%)	162 (26%)	161 (31%)
Not prepared	870 (74%)	470 (75%)	400 (74%)	814 (72%)	459 (74%)	355 (69%)
Unknown	83	36	47	50	29	21
Wish of life-prolongation treatment	41 (3.4%)	22 (3.4%)	19 (3.4%)	39 (3.4%)	25 (4.0%)	14 (2.7%)

Depressive symptoms were associated with a higher likelihood of ACP discussion (adjusted PR, 1.20; 95% confidence interval [CI], 1.05-1.37) (Table 2). Depressive symptoms were not associated with an increased choice of life-extending treatment (adjusted PR, 1.01; 95% CI, 0.99-1.02).

**Table 2:** Association Between CES-D Scores and the Prevalence of Each Endpoint [Prevalence Ratio (95% CI)] in the Cross-sectional, Multiple-imputed Analysis

CES-D score	Endpoints	
	ACP discussion	Life-prolonging treatment wishes
≤9	Reference	Reference
≥10	1.20 (1.05-1.37)	1.01 (0.99-1.02)

### Longitudinal Analysis

The baseline results by CES-D are presented in Table 3. The incidence of ACP discussion was 18% (118/870) for 1 year.

**Table 3:** Baseline Patient Characteristics for the Longitudinal Analysis, Summarized by Baseline CES-D Score Groups

Patient characteristics	All	CES-D score	
		≤ES	≥ES
	n = 870	n = 470	n = 400
Age			
≤54 y	187 (21%)	96 (20%)	91 (23%)
55-64 y	237 (27%)	127 (27%)	110 (28%)
65-74 y	302 (35%)	168 (36%)	134 (34%)
75-84 y	129 (15%)	70 (15%)	59 (15%)
≥85 y	15 (1.7%)	9 (1.9%)	6 (1.5%)
Gender			
Female	258 (30%)	130 (28%)	128 (32%)
Household income			
<3 M yen	395 (45%)	188 (40%)	207 (52%)
3-5 M yen	186 (21%)	123 (26%)	63 (16%)
5-8 M yen	79 (9.1%)	50 (11%)	29 (7.2%)
>8 M yen	66 (7.6%)	47 (10%)	19 (4.8%)
Unknown	144 (17%)	62 (13%)	82 (20%)
Marital status			
Never married	154 (18%)	75 (16%)	79 (20%)
Married	546 (63%)	305 (65%)	241 (60%)
Widowed	83 (9.5%)	45 (9.6%)	38 (9.5%)
Divorced/separated	72 (8.3%)	37 (7.9%)	35 (8.8%)
Unknown	15 (1.7%)	8 (1.7%)	7 (1.8%)
Educational background			
Less than 12 y	215 (25%)	100 (21%)	115 (29%)
High school	351 (40%)	191 (41%)	160 (40%)
College or university	101 (12%)	64 (14%)	37 (9.2%)
Graduate school	180 (21%)	109 (23%)	71 (18%)
Unknown	23 (2.6%)	6 (1.3%)	17 (4.2%)
Duration of hemodialysis			
<1.0 y	194 (22%)	116 (25%)	78 (20%)
1.0-5.0 y	300 (34%)	145 (31%)	155 (39%)
5.0-10.0 y	155 (18%)	91 (19%)	64 (16%)
>10.0 y	221 (25%)	118 (25%)	103 (26%)
Comorbid condition			
Cardiovascular diseases	407 (47%)	208 (44%)	199 (50%)
Cerebrovascular diseases	150 (17%)	76 (16%)	74 (18%)
Respiratory diseases	58 (6.7%)	23 (4.9%)	35 (8.8%)
Cancers	99 (11%)	54 (11%)	45 (11%)
Psychiatric diseases	31 (3.6%)	10 (2.1%)	21 (5.2%)
ACP status			
ACP discussion	136 (20%)	73 (20%)	63 (22%)
Not prepared	531 (80%)	298 (80%)	233 (78%)
Unknown	203	99	104

Abbreviations: AD, Advance directive.

Depressive symptoms did not appear to be associated with an increased incidence of ACP discussion (adjusted IPR, 1.10; 95% CI, 0.80-1.51) (Table 4). Depressive symptoms were not associated with an increased choice of life-extending treatment (adjusted IPR, 1.03; 95% CI, 0.999-1.06).

**Table 4:** Association Between CES-D Scores and the Incidence of Each Endpoint (Incidence Proportion Ratio (95% CI)) in the Longitudinal, Multiple-imputed Analysis

CES-D score	Endpoints	
	ACP discussion	Life-prolonging treatment wishes
≤9	Reference	Reference
≥10	1.10 (0.80-1.51)	1.03 (0.999-1.06)

## Discussion

We investigated the association between depressive symptoms, as measured using the CES-D, and ACP in Japanese patients receiving HD enrolled in J-DOPPS. The results showed that 26%-28% of patients receiving HD engaged in ACP discussions. Our cross-sectional analysis showed that patients with depression receiving HD exhibited a higher prevalence of engaging in ACP discussions than those without depression. However, in the longitudinal analysis, there was no statistically significant higher incidence of ACP discussion among patients with depressive symptoms receiving HD. Both the cross-sectional and longitudinal analyses showed no significant associations between depressive symptoms and preference for life-prolonging therapy.

Our finding that 26%-28% of patients receiving HD discussed ACP decreases within the range of previously reported prevalence rates of ACP in patients with KFRT. Variation in the prevalence depends on the definition of ACP, sampling method, legal and cultural background of the region, and survey timing (especially before and after the coronavirus disease (COVID)-19 pandemic). In the United States, roughly 10%-30% of individuals engage in ACP, but the rate decreases to approximately 3% among those aged below 65 years.^27^, ^28^, ^29^ The proportion of ACP preparedness among Japanese HD patients between 2021 and 2023 ranged from 11% to 26%, which is similar to or lower than our ACP discussion rates obtained before the COVID-19 pandemic.^6^^,^^30^

The cross-sectional association between depressive symptoms and a higher likelihood of ACP discussion in our study is consistent with the results in the primary care setting. Based on previous literature, 4 potential pathways have been proposed to explain how depressive symptoms may be associated with ACP and EoL discussions: (1) an increased sense of burden on family members, (2) a shift in values from life prolongation to pain relief because of chronic illness, (3) depression-related wishes to discontinue dialysis, and (4) more frequent medical encounters prompted by depression.^31^, ^32^, ^33^, ^34^ This notion is supported by previous research indicating that depressive symptoms can foster future discussions about EoL care under certain circumstances.^35^ Our cross-sectional association may suggest that discussion of EoL care between patients receiving HD and their family members may predispose them to depressive moods. However, we believe the possibility of the reverse causation is low because a systematic review found that ACP reduced depressive symptoms in patients and their families.^36^

The failure to demonstrate an association between depressive symptoms and the occurrence of ACP discussion may be attributed to several reasons as study limitations. First, an insufficient sample size may result in a loss of power. Because the positive point estimate of the IPR of the ACP discussion is consistent with the positive relative risks obtained from previous studies and the present cross-sectional analysis, it may be possible to demonstrate that depressive symptoms may increase ACP involvement through larger sample size studies.^9^ Second, health literacy and spirituality may serve as negative confounders in the association between depressive symptoms and ACP. Because health literacy promotes ACP and reduces depressive symptoms, patients without depressive symptoms would be expected to have higher health literacy than those with depressive symptoms.^37^ Adjustments of spirituality have been suggested to strengthen the magnitude of the association between depressive symptoms and ACP.^9^ The direction in which religious beliefs confound the association between depressive symptoms and ACP may depend on cultural and religious type. In Taiwan and Japan, religious beliefs have been found to facilitate ACP discussions, whereas the findings are controversial in Western countries.^5^^,^^38^, ^39^, ^40^ Large-scale studies in the United States have shown that high levels of religious activity and belief reduce the risk of depression and promote recovery, and similar trends have also been observed in small-scale data on hemodialysis patients.^40^, ^41^, ^42^ However, it has also been shown that these effects are influenced by generation and culture.^43^^,^^44^ In contrast, a positive association between religiousness and depressive symptoms was reported in Japan.^45^^,^^46^ Accordingly, whether spiritual adjustment strengthens the association between depressive symptoms and ACP should also be examined among patients receiving HD. Third, population-specific characteristics may have influenced our results. For example, Japanese culture places a significant emphasis on the family in ACP discussions; however, our study did not measure family-related variables.^22^^,^^47^^,^^48^

The aforementioned limitations may also apply to the failure to demonstrate an association between depressive symptoms and desire for life-extending treatment. A previous study among the general older population suggested a weak positive association between depressive symptoms and refusal of life-extending treatment.^49^ The possibility that mental confusion caused by depressive symptoms may lead to inappropriate decisions about receiving life-extending treatment, ie, patients' involuntary decisions about life-extending treatment, warrants further study.

This study has several strengths. First, we investigated the association between depressive symptoms, measured using an established scale, and ACP in patients undergoing HD. Second, representativeness was ensured by employing a 2-stage random sampling procedure in the J-DOPPS dataset.

In conclusion, we found an association between depressive symptoms and a greater likelihood of ACP discussion in a cross-sectional analysis, but not in a longitudinal analysis. Further research is warranted to understand the factors that promote ACP of the patient's true intention while reducing depressive symptoms.
